# Editorial: Experimental and computational methods in the development of diagnostics and therapeutics for colon cancer

**DOI:** 10.3389/fmolb.2025.1568721

**Published:** 2025-02-19

**Authors:** Muhammad Suleman, Shahid Ali, Farooq Rashid, Faez Iqbal Khan

**Affiliations:** ^1^ Centre for Biotechnology and Microbiology, University of Swat, Mingora, Pakistan; ^2^ Laboratory of Animal Research Center (LARC), QU Health, Qatar University, Doha, Qatar; ^3^ Department of Biotechnology, Guangxi Veterinary Research Institute, Nanning, China; ^4^ Department of Biosciences and Bioinformatics, School of Science, Xi’an Jiaotong-Liverpool University, Suzhou, China

**Keywords:** colon cancer, diagnostics, therapeutics, machine-learning, computational, experimental

Cancer continues to be one of the major causes of illness and death worldwide. It is growing an alarming rate, and affects every geographic region of the world. Therefore, the goal is to investigate novel biomarkers both experimentally and computationally in the development of colon cancer and drug resistance. Computational methods play a crucial role in modern cancer research, facilitating drug discovery and improving therapeutic strategies (Ahmad et al., 2024; Shaikh et al., 2023). In this Research Topic, we aim to provide an overview of recent technologies in experimental and computational areas, such as artificial intelligence and machine learning approaches, relevant to the identification of novel biomarkers and drug testing in cancer diagnosis, management, and treatment. Recent studies highlight three key areas: biomarker discovery, therapeutic developments, and computational modeling in cancer research. Grouping the studies under these themes provides a cohesive narrative, highlighting key trends and challenges in the field.

The identification of novel biomarkers is crucial for early detection and targeted therapy in cancer treatment. Sorokin et al. compared gene fusion detection in colorectal cancer patients, identifying 93 new fusion genes, with 11 appearing in multiple patients. Notably, a novel LRRFIP2-ALK fusion was identified, with potential implications for ALK inhibitor therapies. Cao et al. developed a prognostic model using three cellular senescence-related lncRNAs (CSRLs), demonstrating its predictive power for overall survival and immune response in colon cancer patients. Tambaro et al. analyzed circulating miRNAs in gastrointestinal (GI) cancer patients, finding specific miRNAs associated with adiposity levels and cachexia, suggesting their potential as diagnostic biomarkers. Wang et al. explored the prognostic and immunogenic characteristics of disease regulators in colon cancer. Their analysis revealed that age, tumor stage, and ferroptosis score were significantly correlated with prognosis.

Understanding drug resistance and optimizing therapeutic strategies are crucial for effective cancer treatment. Burov et al. investigated the effects of multikinase inhibitors (MKIs) on proteasome expression in colorectal cancer cells, revealing increased expression of non-constitutive proteasomes in BRAF-mutant tumors. MKI treatment induced oxidative stress and proteasome redistribution, shedding light on potential therapeutic targets. Yang et al. developed a necroptosis-related prognostic model using key genes (CALB1, CHST13, and SLC4A4), effectively predicting patient survival and immune response. Bukhari et al. discussed the clinical implications of long noncoding RNA LINC-PINT, emphasizing its role as a potential therapeutic target in cancer treatment. Zhang et al. reviewed the impact of immunosenescence on solid gastrointestinal tumors, highlighting how senescence-related mechanisms diminish immunotherapy effectiveness and proposing senotherapy strategies to counteract these effects.

Computational models enhance predictive accuracy and statistical analyses in cancer research. Su et al. compared competing-risk analysis and Cox regression models to assess prognostic factors in adenocarcinoma of the transverse colon (ATC), finding that traditional Cox regression underestimated cancer stage-related risks. Prior studies on AI-driven drug discovery, such as sphingosine kinase 1 inhibitors and FDA-approved PIM-1 kinase inhibitors, demonstrate the potential of virtual screening and molecular dynamics simulations in identifying promising therapeutic compounds (Khan et al., 2020; Rathi et al., 2024). Wang et al. investigated the PD-1/PD-L1 interaction mechanism using computational approaches, reinforcing the role of immune checkpoint inhibitors like pembrolizumab in cancer treatment (Wang and Khan, 2023). Insights from previous studies on human peroxiredoxin 6 further underscore the intricate roles of molecular targets in cancer, demonstrating its dual role in cancer progression and inhibition, highlighting its potential as a therapeutic target (Qausain et al., 2023).

This Research Topic underscores the significant advancements in biomarker discovery, therapeutic development, and computational modeling in cancer research. The integration of AI and ML continues to enhance predictive accuracy, streamline drug discovery, and improve patient outcomes. Despite these advancements, challenges remain in overcoming drug resistance and improving the efficacy of immunotherapies. Future research should refine computational models, explore novel therapeutic targets, and bridge experimental and computational methodologies to optimize cancer diagnosis and treatment.
